# A Novel Ashwagandha (*Withania somnifera*) Formulation Mitigates Sleep Deprivation-Induced Cognitive Impairment and Oxidative Stress in a Rat Model

**DOI:** 10.3390/biom15050710

**Published:** 2025-05-12

**Authors:** Besir Er, Busra Ozmen, Emre Sahin, Cemal Orhan, Nurhan Sahin, Abhijeet A. Morde, Muralidhara Padigaru, Kazim Sahin

**Affiliations:** 1 Department of Biology, Faculty of Science, Firat University, Elazig 23119, Turkey; ber@firat.edu.tr; 2 Department of Animal Nutrition, Faculty of Veterinary Medicine, Firat University, Elazig 23119, Turkey; busraagzikucuk01@gmail.com (B.O.); corhan@firat.edu.tr (C.O.); nsahin@firat.edu.tr (N.S.); 3 Department of Animal Nutrition, Faculty of Veterinary Medicine, Bingol University, Bingol 12100, Turkey; esahin@bingol.edu.tr; 4 Research and Development, OmniActive Health Technologies Co., Ltd., Mumbai 400013, India; a.morde@omniactives.com (A.A.M.); m.padigaru@omniactives.com (M.P.)

**Keywords:** ashwagandha, withanolides, memory, sleep deprivation, stress, GABAergic pathway

## Abstract

Ashwagandha (*Withania somnifera*) is a well-known adaptogenic herb traditionally used to enhance sleep quality and mitigate stress-induced cognitive decline. This study investigated the effects of different doses of ashwagandha root extract (AE) formulations on cognitive function, oxidative stress, and neuronal plasticity in a rat model of sleep deprivation (SD). Forty-nine rats were randomly assigned to seven groups: control, wide platform (WP), SD, SD + A1 (15 mg/kg AE 1.5%), SD + A2 (30 mg/kg AE 1.5%), SD + A3 (5.5 mg/kg AE 8.0%), and SD + A4 (11 mg/kg AE 8.0%). The extract was administered orally for four weeks. SD induced via a modified wide platform model significantly impaired spatial memory, increased oxidative stress, and suppressed GABA receptor activity. Treatment with all AE doses, except 15 mg/kg AE 1.5%, considerably reduced serum corticosterone (12% for SD + A2, 15% for SD + A3, and 32% for SD + A4), CRH (11% for SD + A2, 14% for SD + A3, and 17% for SD + A4), ACTH (22% for SD + A2, 26% for SD + A3, and 38% for SD + A4), and MDA levels (31% for SD + A2, 34% for SD + A3, and 46% for SD + A4) (*p* < 0.05). All doses improved antioxidant enzyme activity and memory performance, while AE 8.0% doses notably increased serotonin (19% for SD + A3 and 33% for SD + A4) and dopamine levels (40% for SD + A3 and 50% for SD + A4). Moreover, AE treatment enhanced markers of neuronal plasticity and partially improved GABAergic function. These findings suggest that AE formulations, particularly at higher concentrations, exert neuroprotective effects against SD-induced cognitive impairment by modulating oxidative stress, neurotransmitter balance, and neuroplasticity, indicating their potential application in managing stress-related neurological disorders.

## 1. Introduction

Cognitive health depends on a complex interplay of physiological and neurological processes, with sleep playing a pivotal role in maintaining memory and brain function [1]. Sleep deprivation (SD)—whether due to insufficient duration or poor quality—is increasingly prevalent in modern society and is known to impair various aspects of cognition [2]. Evidence from both human and animal studies indicates that SD detrimentally affects motor performance, cognitive processing, and emotional regulation, often exacerbating anxiety and depressive behaviors [3]. Moreover, sleep disturbances such as insomnia and fragmented sleep are frequently observed in patients with neurodegenerative diseases like Alzheimer’s, as well as in aging animals with cognitive dysfunction [4]. Therefore, elucidating the intricate molecular and hormonal relationship between cognitive function, sleep disturbance, and stress is vital for developing effective interventions to mitigate the adverse effects of SD-related stress for humans and animals [3,5].

SD is closely linked to the activation of the hypothalamic–pituitary–adrenal (HPA) axis, leading to elevated levels of corticotropin-releasing hormone (CRH) and adrenocorticotropic hormone (ACTH), which can interfere with memory consolidation [6,7]. Additionally, SD induces oxidative stress by reducing total antioxidant capacity and the activity of critical enzymes such as superoxide dismutase (SOD), catalase (CAT), and glutathione peroxidase (GSH-Px), thereby increasing neuronal vulnerability [8].

Alterations in neurotransmitter systems—particularly serotonin, dopamine, and gamma-aminobutyric acid (GABA)—also disrupt synaptic function and neuroplasticity, essential components of memory encoding and retrieval [9]. Neuroplasticity and cellular communication markers, such as the neural cell adhesion molecule (NCAM) [10], intercellular adhesion molecule-1 (ICAM-1) [11], brain-derived neurotrophic factor (BDNF), and nerve growth factor (NGF), are also adversely affected, impairing synaptic connectivity and plasticity in sleep-deprived states [12]. It possibly mitigates stress and detrimental effects of SD by regulating cortisol release [13] and protein expression of GABA_A_, GABA_B_1, and serotonin receptors in the brain [14].

Ashwagandha (*Withania somnifera*) is a medicinal plant widely recognized for its therapeutic properties, primarily attributed to a group of naturally occurring compounds known as withanolides. These steroidal lactones, predominantly found in the roots and, to a lesser extent, in the leaves and stems, possess a C28 ergostane backbone with a lactone ring structure. In addition to withanolides, the plant contains other bioactive components such as withaferin A, withanone, alkaloids, and steroidal lactones, which contribute to its broad pharmacological potential. Major identified withanolides include withanolide A–D, withanoside IV and V, withanone, withaferin A, and sitoindosides VII–X [15]. The ashwagandha extract (AE) used in this study is a standardized water–alcoholic preparation containing 1.5% total withanolides, quantified by high-performance liquid chromatography (HPLC) in accordance with the United States Pharmacopeia (USP) guidelines, which define seven specific peaks for withanolide analysis [16]. This standardized method ensures consistency and reliability, addressing limitations found in earlier studies that often relied on non-standardized commercial extracts with variable and unverified withanolide content.

Withanolides have been extensively studied for their diverse biological effects. These include antioxidant activity through the reduction in oxidative stress, anti-inflammatory effects via modulation of cytokine pathways, immunomodulatory actions that enhance or balance immune responses, and neuroprotective effects that support neuronal health and function. Moreover, several withanolides have shown promising antitumor activity and have been linked to cognitive enhancement and stress resilience [17,18]. Ashwagandha extract (AE) is a natural sleep-promoting agent known for its favorable safety profile and efficacy in improving sleep onset and quality in individuals with insomnia. Its beneficial effects are thought to involve modulation of GABAergic pathways and brain-derived neurotrophic factor (BDNF), contributing to reduced stress and preserved neuroplasticity [17,18]. However, the poor solubility of ashwagandha’s key bioactive components—particularly polar withanolide glycosides—may limit its bioavailability and clinical effectiveness [19]. Furthermore, the dose-dependent effects of AE on SD-related cognitive and molecular changes remain insufficiently understood.

This study aimed to investigate the potential neuroprotective effects of different doses of a water-soluble, highly bioavailable AE formulation on memory impairment induced by SD in a rat model. We focused on evaluating biochemical and molecular markers of oxidative stress [e.g., malondialdehyde (MDA), antioxidant enzymes], hormonal responses (e.g., corticosterone, CRH, ACTH), and neural plasticity including BDNF, NGF, NCAM, and growth-associated protein 43 (GAP-43), as well as the expression of GABAergic receptor subtypes (GABA_A_R2, GABA_B_R1, GABA_B_R2) in the brain. This comprehensive approach aimed to clarify the dose-dependent impact of AE on SD-related stress and cognitive dysfunction.

## 2. Materials and Methods

### 2.1. Animals and Experimental Design

A total of 49 male Sprague–Dawley rats (n = 7), 8 weeks old, were used in this study. The animals were housed under standard laboratory conditions, including a controlled temperature of 22 ± 2 °C, relative humidity of 55 ± 5%, and a 12 h light/dark cycle. All rats had ad libitum access to food and water. The experimental protocol was approved by the Animal Ethics Committee of Fırat University (Approval No.: 06.05.2024-23993) and conducted in accordance with the National Institutes of Health Guide for the Care and Use of Laboratory Animals and relevant EU directives (approval date 6 May 2024).

**Figure 1:**
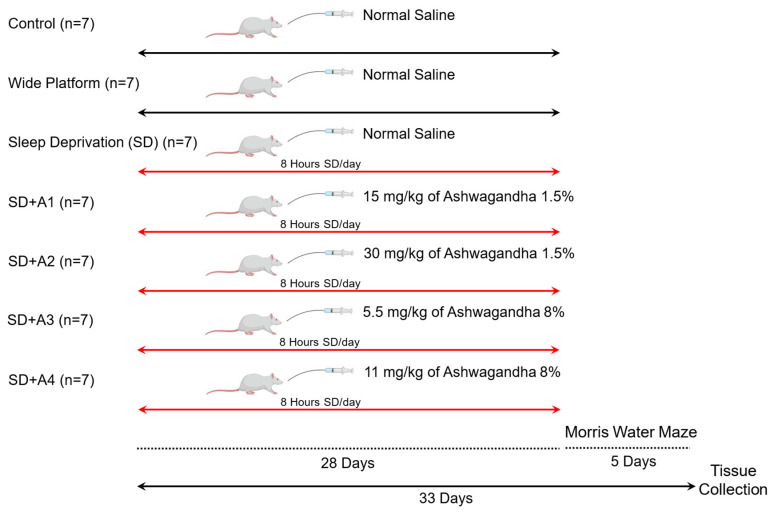
Experimental procedures.

The rats were randomly assigned into seven groups (n = 7 per group), each designed to assess the effects of SD and AE at different concentrations and dosages: (1) Control (C): Rats were maintained under standard conditions without exposure to SD. They received daily oral gavage of normal saline throughout the experimental period. (2) Wide platform (WP): This group served as a procedural control for the SD setup. Rats were placed on large platforms that allowed for normal sleep while controlling for the environmental stress of platform housing. They also received daily oral saline. (3) Sleep deprivation (SD): Rats were subjected to SD for 8 h per day (08:30–16:30) for four weeks using a modified multiple-platform method to induce stress. These rats received oral saline daily and served as the negative control group. (4) SD + A1: Rats were sleep-deprived as in the SD group and treated with 15 mg/kg of ashwagandha 1.5% extract (low dose of standard concentration) via oral gavage once daily for four weeks. (5) SD + A2: Rats were sleep-deprived and received a higher dose of ashwagandha 1.5% extract (30 mg/kg) daily to assess potential dose-dependent effects of the standard concentration formulation. (6) SD + A3: This group was sleep-deprived and treated with a low dose (5.5 mg/kg) of a concentrated ashwagandha 8.0% extract formulation to examine the efficacy of a more potent preparation. (7) SD + A4: Sleep-deprived rats in this group received 11 mg/kg of the ashwagandha 8.0% extract (high dose of the concentrated formulation). All treatments were administered via oral gavage once daily for four weeks, starting simultaneously across all groups (Figure 1).

Samples of AE containing 1.5% (item code: 301273) and 8% (item code: 301274) withanolides, the active phytochemical constituents, were obtained from OmniActive Health Technologies Ltd. (Thane, India). These formulations are designed for improved solubility and bioavailability, utilizing cellulose polymers as hydrophilic carriers. The varying concentrations of *Withania somnifera* extract were incorporated into the formulations to create water-dispersible products suitable for oral administration in experimental settings.

### 2.2. Induction of Sleep Deprivation

**Figure 2:**
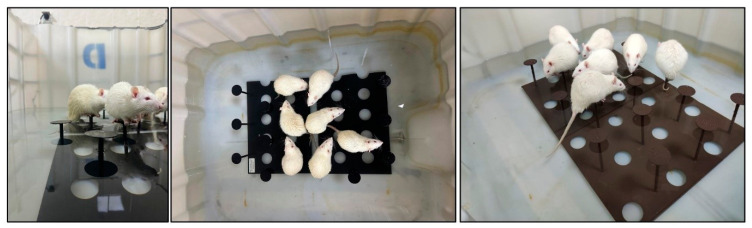
Sleep deprivation (SD) platform images for rats.

SD was induced using multiple columns in the modified platform (in water) model [20]. Rats were positioned in a large tank measuring 170 cm in length, 40 cm in width, and 55 cm in depth, which contained tap water at a temperature of 24 °C. The tank included 20 small platforms, each with a diameter of 5 cm, positioned 10 cm apart from edge to edge and organized into two rows (Figure 2). The tank contained water up to 2 cm under the platform surface. In this tank, animals were able to traverse freely between platforms. Muscle atonia occurred during the paradoxical sleep period [21], causing the animals to fall into the water and wake up. Following that, they ascended to the platform and seated themselves upon it again. To determine the effects of possible stresses that may occur in the tank environment, WP with a diameter of 12 cm was used to ensure that the rats slept uninterruptedly and to prevent them from falling into the water.

### 2.3. Morris Water Maze

The Morris water maze (MWM) test was used to test spatial learning and memory among all groups of animals at the end of the study for 5 days. This model and the detailed procedure were previously described [22]. A circular platform with a diameter of 10 cm was positioned at the center of one quadrant of the tank, situated 1.5 cm below the water’s surface. Initially, the platform was discreetly placed in a randomly selected area of the reservoir to ensure its concealment throughout the testing period. The pool was divided into four equal quadrants by the designated starting positions (north, south, east, and west) arranged along its perimeter. Each rat was allowed a maximum of 60 s to locate the submerged platform and was required to remain on it for 30 s. In instances where a rat was unable to find the hidden platform within this timeframe, it was gently placed on the platform for a duration of 10 s. Following this, the rat was returned to a heated cage for a five-minute recuperation period between trials and subsequently released into the water from a different starting point to repeat the procedure. Upon completion of the fifth day of trials, a probe test was conducted. During this assessment, the platform was removed from the water, and the rats were positioned at the center of the tank to monitor their movements for 60 s. The duration each rat spent in proximity to the area where the platform was previously located was recorded and analyzed.

### 2.4. Sample Collection

All rats were overnight-fasted after the MWM test (33rd day), and blood was taken from decapitated animals through cervical dislocation. Blood samples were collected in biochemical tubes, and the serum samples were subsequently subjected to centrifugation at 4 °C with a force of 2300× *g* for 10 min in a refrigerated centrifuge. Brain tissues were promptly extracted and stored in a deep freezer at −80 °C until their analysis was conducted.

### 2.5. Biochemical Analysis

The levels of serum biochemical markers such as serum glucose, cholesterol, triglycerides, blood urea nitrogen (BUN), and creatinine and the activities of aspartate aminotransferase (AST) and alanine aminotransferase (ALT) were assessed using a portable automated chemistry analyzer (Samsung LABGEO PT10, Samsung Electronics Co., Suwon, Republic of Korea) with rat-specific kits (IVR-PT06, Samsung LABGEO PT Biochemistry Test 15, Samsung Electronics Co., Suwon, Republic of Korea).

Corticosterone, CRH, ACTH (Cayman Chemical Co., Ann Arbor, MI, USA), serotonin, dopamine (Elabscience Biotechnology, Wuhan, China), total antioxidant activity (TAC), and antioxidant enzymes (SOD, CAT, GSHPx) were quantified using appropriate commercial kits (BT-LABS, Shanghai, China) following the manufacturer’s instructions in a microplate reader (Elx-800, Bio-Tek Instruments Inc., Winooski, VT, USA).

The levels of serum, liver, and brain MDA were assessed using a high-performance liquid chromatography system (HPLC, Shimadzu, Kyoto, Japan). A system incorporating a UV–vis SPD-10 AVP detector and a CTO-10 AS VP column was chosen for the analysis. The mobile phase consisted of a mixture of 30 mM KH_2_PO_4_ and methanol (82.5:17.5, *v*/*v*, pH 3.6), with a flow rate established at 1.2 mL/min.

### 2.6. Western Blot Analysis

Brain samples were homogenized in a cold Tris-HCl buffer (10 mM, pH 7.4) with the addition of protease inhibitors (Sigma, St. Louis, MO, USA). Following homogenization, the samples were centrifuged at 15,000× *g* for 30 min at 4 °C. The supernatants obtained were then heated and mixed with 2x Laemmli sample buffer to prepare for analysis. To facilitate the separation of proteins, the Mini-PROTEAN Tetra Cell system (Bio-Rad, Hercules, CA, USA) was utilized for the 12% SDS-PAGE stage. Subsequently, proteins were transferred to nitrocellulose membranes via a semi-dry transfer method using a Power Blotter (Thermo Fisher, Waltham, MA, USA). The membranes underwent an incubation with a 5% bovine serum albumin solution at room temperature for two hours to block non-specific binding to proteins. They were then incubated overnight at 4 °C with antibodies specific to the brain, including NCAM, BDNF, NGF, GAP-43, GABA_A_R2, GABA_B_R1, GABA_B_R2, and 5-HT1A (Santa Cruz Biotechnology, Dallas, TX, USA). Following the blocking and incubation phase, membranes were treated with an HRP-linked secondary antibody at room temperature for two hours. To assess the presence of proteins, an anti-β-actin antibody (Sigma, St. Louis, MO, USA) was employed, followed by the application of the diaminobenzidine substrate method to observe antibody interactions. Densitometric analyses of the protein bands were conducted using ImageJ software (version 1.54g, National Institutes of Health, Bethesda, MD, USA).

### 2.7. Statistical Analysis

The SPSS statistical package program (IBM SPSS Version 22.0) was used for data analysis. The sample size (N = 49) was determined using the G*Power program (Version 3.1.9.3) with a power of 85%, an effect size of 0.65, and a significance level of 0.05. All data are presented as the mean ± standard error of the mean (SEM). A one-way analysis of variance (ANOVA), followed by the Tukey post hoc test, was employed for multiple comparisons of parametric data. Conversely, non-parametric data (probe trial) were analyzed using the Kruskal–Wallis and Mann–Whitney U tests. The threshold for statistical significance was established at *p* < 0.05.

## 3. Results

### 3.1. Body Weight and Serum Biochemical Parameters

Rats exposed to chronic stress induced by SD showed a significant reduction in final body weight compared to both the control and WP groups (*p* < 0.001). Among the ashwagandha-treated groups, only the highest dose of the 8% extract (11 mg/kg; SD + A4 group) significantly prevented this weight loss, resulting in a final body weight (BW) that was higher than that in the SD group (*p* < 0.05), though still lower than the control group.

Chronic stress led to increased serum glucose and AST levels in the SD group compared to healthy control rats (*p* < 0.001). Notably, the higher dose of AE at 11 mg/kg (8% ashwagandha) decreased serum glucose and AST levels in comparison to those in the SD group (*p* < 0.01). We observed that serum BUN levels decreased due to stress induced by SD (*p* < 0.001), and only the higher dose of AE at 11 mg/kg of 8% ashwagandha was effective in partially reversing this decrease (*p* < 0.01) (Table 1).

### 3.2. Serum Hormone Levels

**Figure 3:**
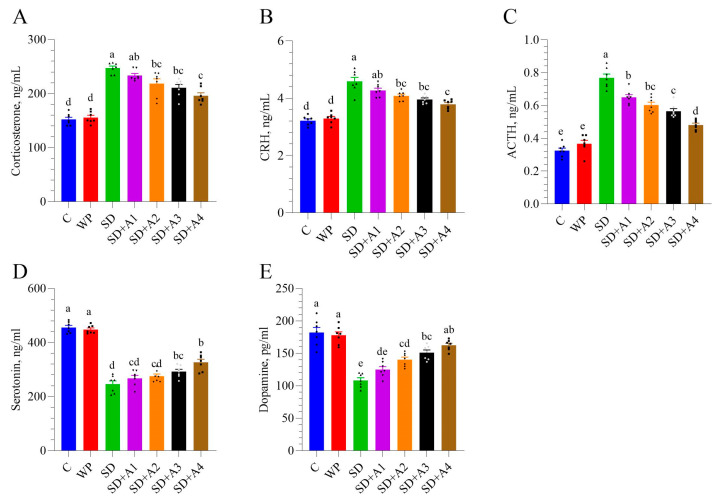
Effects of different ashwagandha doses on corticosterone (**A**), CRH (**B**), ACTH (**C**), serotonin (**D**), and dopamine (**E**) in sleep-deprived rats. Data were analyzed using one-way ANOVA followed by Tukey’s post hoc test for multiple comparisons. Different superscript letters (a–e) indicate statistically significant differences between groups (*p* < 0.05). The groups not sharing the same letter are significantly different. The error lines indicate SEM, and symbols show individual values. CRH: corticotropin-releasing hormone, ACTH: adrenocorticotropic hormone, C: control, WP: wide platform, SD: sleep deprivation, SD + A1: SD + ashwagandha 1.5% (15 mg/kg), SD + A2: SD + ashwagandha 1.5% (30 mg/kg), SD + A3: SD + ashwagandha 8% (5.5 mg/kg), SD + A4: SD + ashwagandha 8% (11 mg/kg).

SD significantly elevated serum levels of stress-related hormones, including corticosterone (panel A), CRH (panel B), and ACTH (panel C), compared to both the control and WP groups (*p* < 0.001, Figure 3). These increases confirm the successful induction of chronic stress through SD. Treatment with AE effectively modulated these stress markers. Specifically, 30 mg/kg of ashwagandha 1.5% (SD + A2, *p* < 0.01) and both doses of ashwagandha 8% (SD + A3 and SD + A4, *p* < 0.001 for all) significantly reduced corticosterone, CRH, and ACTH levels compared to those in the untreated SD group. Notably, the 11 mg/kg dose of ashwagandha 8% (SD + A4) demonstrated the most pronounced effect, bringing hormone levels closer to those of the control group. Furthermore, even the low dose of ashwagandha 1.5% (SD + A1) significantly reduced ACTH levels, although its impact on corticosterone and CRH was limited.

In terms of neurotransmitters, SD led to a significant decline in serum serotonin (panel D) and dopamine (panel E) levels, indicating disrupted mood and cognitive signaling. AE treatment, especially at higher doses, ameliorated these effects (Figure 3). Serotonin levels were partially improved by both doses of ashwagandha 8% (*p* < 0.05 for SD + A3 and *p* < 0.001 for SD + A4), while dopamine levels were significantly enhanced in the SD + A2, SD + A3, and SD + A4 groups (*p* < 0.001). Notably, the rats receiving 11 mg/kg ashwagandha 8% had serum dopamine levels similar to those of the control group rats (*p* > 0.05).

### 3.3. Malondialdehyde and Antioxidant Enzymes

**Figure 4:**
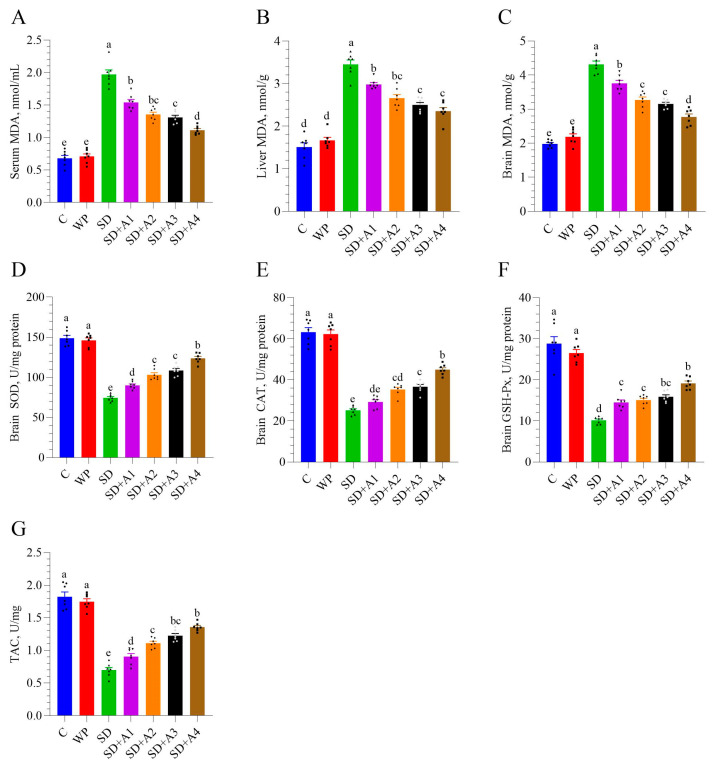
Effects of different ashwagandha doses on oxidative stress markers in sleep-deprived rats. (**A**) serum MDA, (**B**) liver MDA, (**C**) brain MDA, (**D**) brain superoxide dismutase (SOD), (**E**) brain catalase (CAT), (**F**) brain glutathione peroxidase (GSH-Px), and (**G**) total antioxidant capacity (TAC). Data were analyzed using one-way ANOVA followed by Tukey’s post hoc test for multiple comparisons. Different superscript letters (a–e) indicate statistically significant differences between groups (*p* < 0.05). The groups not sharing the same letter are significantly different. The error lines indicate SEM, and symbols show individual values. MDA: malondialdehyde, SOD: superoxide dismutase, CAT: catalase, GSH-Px: glutathione peroxidase, TAC: total antioxidant capacity. C: control, WP: wide platform, SD: sleep deprivation, SD + A1: SD + ashwagandha 1.5% (15 mg/kg), SD + A2: SD + ashwagandha 1.5% (30 mg/kg), SD + A3: SD + ashwagandha 8% (5.5 mg/kg), SD + A4: SD + ashwagandha 8% (11 mg/kg).

Chronic stress induced by SD led to a marked increase in MDA levels, a key marker of lipid peroxidation and oxidative stress, in the serum (panel A), liver (panel B), and brain (panel C) (*p* < 0.001, Figure 4). In contrast, the control and WP groups maintained significantly lower MDA levels, confirming the impact of SD on systemic and central oxidative damage. All doses of AE (SD + A1 to SD + A4) reduced serum, liver, and brain MDA levels compared to those in non-supplemented rats (*p* < 0.01, Figure 4). The SD + A4 group showed the most significant decrease in serum and brain MDA levels compared to the other treatments (*p* < 0.001), suggesting dose-dependent protection. Simultaneously, brain antioxidant enzymes (SOD, CAT, GSH-Px) and TAC were suppressed under these stress conditions in rats (*p* < 0.001). All doses of AE (SD + A1 to SD + A4) improved the levels of SOD, CAT, GSH-Px, and TAC in the brain compared to SD group (*p* < 0.05). Notably, the highest dose of AE (11 mg/kg ashwagandha 8%) substantially improved brain SOD and CAT levels compared to other AE formulations (*p* < 0.001). Although the SD + A4 and SD + A3 groups had similar brain GSH-Px and TAC levels (*p* > 0.05), the SD + A4 group exhibited slightly higher values, indicating a more pronounced antioxidant effect at the highest AE dose.

### 3.4. Morris Water Maze

**Figure 5:**
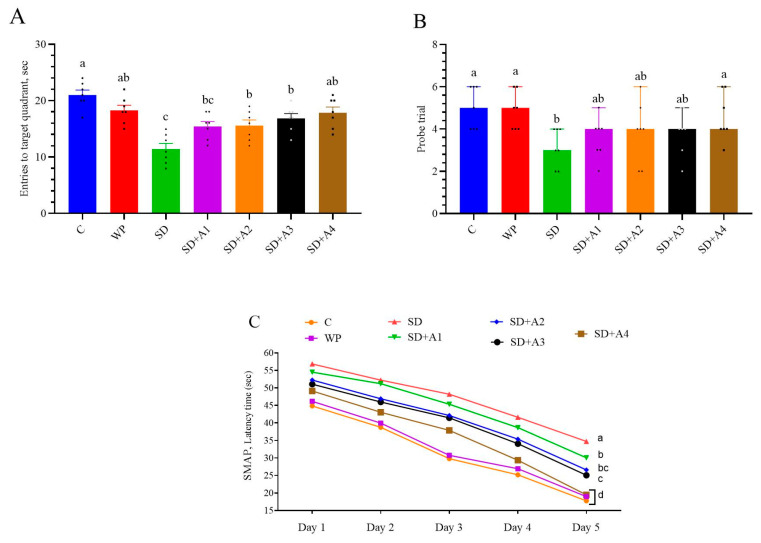
Effects of different ashwagandha doses on entries to target quadrant (**A**), probe trial (**B**), and spatial learning memory acquisition phase (SMAP) (**C**) in sleep-deprived rats. One-way ANOVA was used for group differences, and Tukey post hoc analysis for multiple comparisons. Kruskal–Wallis and Mann–Whitney U tests were used to compare the probe trials. Different superscripts (a–d) indicate the mean differences between the groups (*p* < 0.05). The groups not sharing the same letter are significantly different. The error lines indicate SEM for entries to the target quadrant and 95% confidence interval for the probe trial. Symbols show individual values. C: control, WP: wide platform, SD: sleep deprivation, SD + A1: SD + ashwagandha 1.5% (15 mg/kg), SD + A2: SD + ashwagandha 1.5% (30 mg/kg), SD + A3: SD + ashwagandha 8% (5.5 mg/kg), SD + A4: SD + ashwagandha 8% (11 mg/kg).

The Morris water maze test confirmed that SD-induced stress may impair spatial memory performance. Across all groups, the latency time to reach the target gradually decreased from day 1 to day 5 (Figure 5). On day 5, the latency time was highest in the SD group compared to all groups (*p* < 0.01, except SD + A1), indicating impaired learning ability. All doses of AE showed varying levels of improvement in latency time (*p* < 0.01, except 15 mg/kg ashwagandha 1.5%), and a dose of 11 mg/kg ashwagandha 8% demonstrated the most significant effect, approaching the control and WP groups’ performance (*p* > 0.05). The time of entries to the target quadrant was reduced in the SD group (*p* < 0.001) compared to that in the control and WP groups. Treatment groups SD + A2 (*p* < 0.05), SD + A3 (*p* < 0.01), and SD + A4 (*p* < 0.001) showed significant improvement in the time of entries to the target quadrant as compared to the SD group. Moreover, the time of entries to the target quadrant of the SD + A4 group was similar to the control and WP groups’ levels (*p* > 0.05). The probe trial was not changed between the SD and SD + A1–A3 groups (*p* > 0.05); however, the SD + A4 group showed more probe trials than the untreated SD group (*p* < 0.05).

### 3.5. Brain Neurotrophic Factors

**Figure 6:**
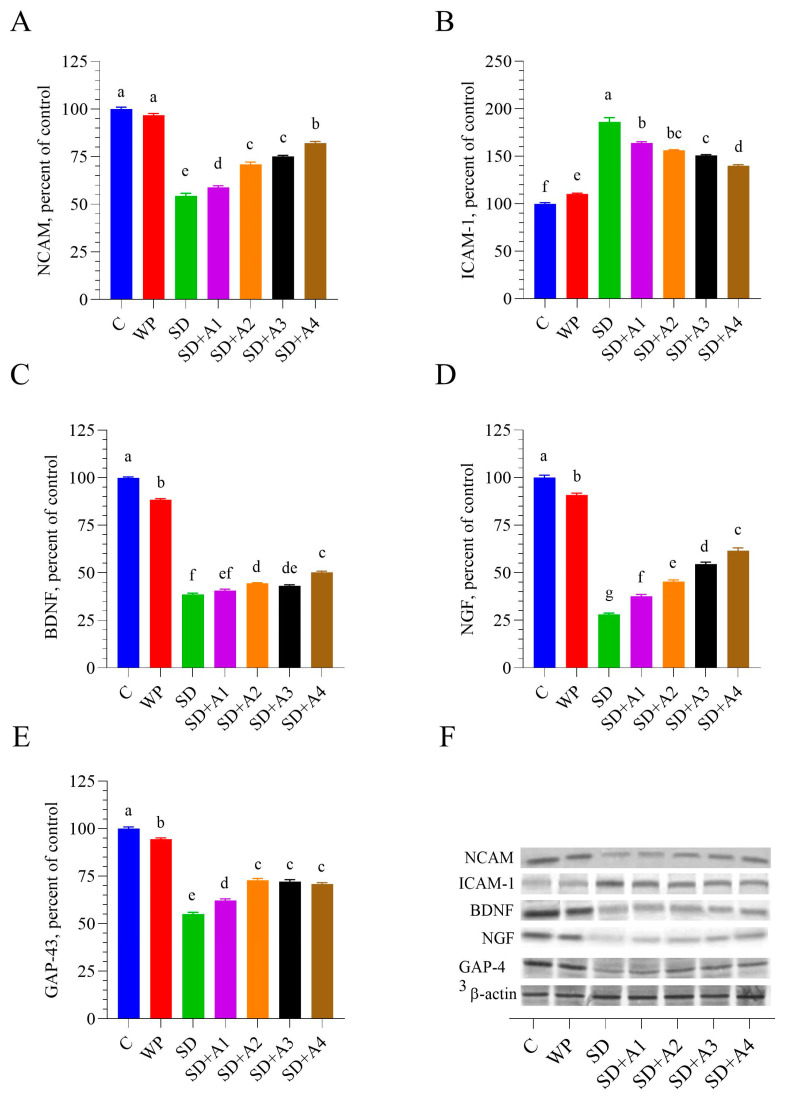
Effects of different ashwagandha doses on brain tissue NCAM (**A**), ICAM-1 (**B**), BDNF (**C**), NGF (**D**), and GAP-43 (**E**) protein levels and representative Western blot bands ((**F**) and Figure S1) in sleep-deprived rats. Western blot analysis was performed with incorporated β-actin to ensure equal protein loading. Representative bands are shown in panel (**F**). Data were analyzed using one-way ANOVA followed by Tukey’s post hoc test for multiple comparisons. Different superscript letters (a–g) indicate statistically significant differences between groups (*p* < 0.05). The groups not sharing the same letter are significantly different. The error lines indicate SEM. NCAM: neural cell adhesion molecule, ICAM: intercellular adhesion molecule-1, BDNF: brain-derived neurotrophic factor, NGF: nerve growth factor. C: control, WP: wide platform, SD: sleep deprivation, SD + A1: SD + ashwagandha 1.5% (15 mg/kg), SD + A2: SD + ashwagandha 1.5% (30 mg/kg), SD + A3: SD + ashwagandha 8% (5.5 mg/kg), SD + A4: SD + ashwagandha 8% (11 mg/kg). The original Western blot image can be found in the Supplementary Materials.

Stress induced by SD significantly decreased brain levels of NCAM, BDNF, NGF, and GAP-43 while simultaneously increasing brain ICAM-1 levels compared to those of healthy control rats (*p* < 0.001). All doses of AE (except for 15 mg/kg ashwagandha 1.5% for BDNF) partially restored the brain activity of NCAM, BDNF, NGF, GAP-43, and ICAM-1 compared to that in the SD group (*p* < 0.05), but 11 mg/kg of ashwagandha 8% formulation demonstrated the most significant partial improvement across all doses (*p* < 0.001, Figure 6). However, the SD + A2–A4 group had similar brain GAP-43 levels (*p* > 0.05).

### 3.6. Brain GABAergic and Serotonergic Receptors

**Figure 7:**
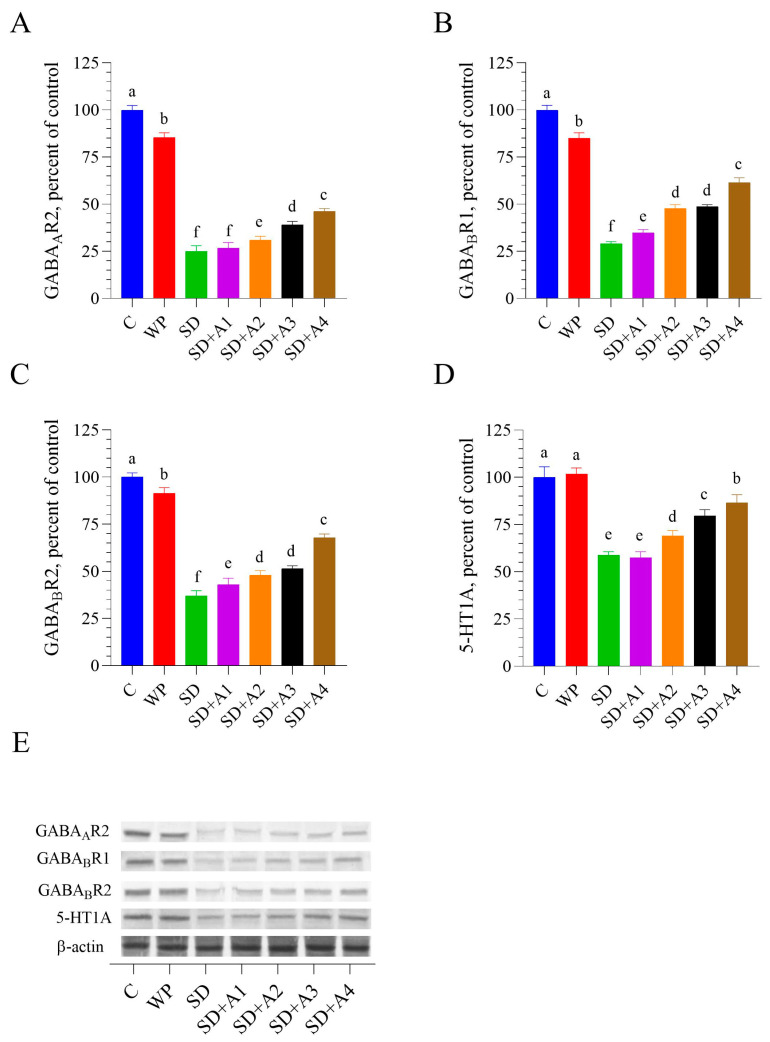
Effects of different ashwagandha doses on brain tissue GABA_A_R2 (**A**), GABA_B_R1 (**B**), GABA_B_R2 (**C**), and 5-HT1A (**D**) protein levels and representative Western blot bands ((**E**) and Figure S2) in sleep-deprived rats. Western blot analysis was performed with incorporated β-actin to ensure equal protein loading. Representative bands are shown in panel E. Data were analyzed using one-way ANOVA followed by Tukey’s post hoc test for multiple comparisons. Different superscript letters (a–f) indicate statistically significant differences between groups (*p* < 0.05). The groups not sharing the same letter are significantly different. The error lines indicate SEM. GABA_A_: gamma-aminobutyric acid type A receptor subunit alpha, GAP-43: growth-associated protein-43. C: control, WP: wide platform, SD: sleep deprivation, SD + A1: SD + ashwagandha 1.5% (15 mg/kg), SD + A2: SD + ashwagandha 1.5% (30 mg/kg), SD + A3: SD + ashwagandha 8% (5.5 mg/kg), SD + A4: SD + ashwagandha 8% (11 mg/kg). The original Western blot image can be found in the Supplementary Materials.

Chronic SD caused a significant reduction in the expression of key neurotransmitter-related receptors in the brain, including GABA_A_R2, GABA_B_R1, GABA_B_R2, and 5-HT1A compared to the control and WP groups (*p* < 0.001, Figure 7). AE treatments demonstrated varying levels of protection against SD-induced suppression, with SD + A2, SD + A3, and SD + A4 showing significant effects compared to the SD group (*p* < 0.01). While the lowest dose (15 mg/kg of ashwagandha 1.5%, SD + A1) failed to impact GABA_A_R2 and 5-HT1A levels (*p* > 0.05 vs. SD), higher doses, namely, 30 mg/kg of ashwagandha 1.5% (SD + A2) and both 5.5 mg/kg (SD + A3) and 11 mg/kg (SD + A4) of ashwagandha 8%, resulted in notable improvement of all four receptors compared to the untreated SD group (*p* < 0.001). Among all treatment groups, SD + A4 (11 mg/kg of ashwagandha 8%) showed the most substantial improvement in the expression of all measured receptors (*p* < 0.05 for the SD + A3, *p* < 0.001 for the SD + A1 and SD + A2 groups for all receptors), indicating a stronger regulatory effect on neurotransmission under chronic stress conditions.

## 4. Discussion

This study comprehensively evaluated the effects of different doses and formulations of AE on physiological, biochemical, and neurobiological parameters in rats exposed to chronic stress induced by SD. The findings demonstrated that AE exerted protective effects across a range of stress-induced impairments, including disruptions in body weight, metabolic biomarkers, oxidative stress, cognitive performance, and neuroplasticity. The most notable improvements were observed with the higher-dose AE formulation (11 mg/kg of 8% extract), suggesting a dose-dependent efficacy consistent with the plant’s adaptogenic and neuroprotective profile. No dose-dependent adverse effects were observed in animals at higher concentrations of AE during the study period. All administered doses were well tolerated, and no signs of toxicity, behavioral abnormalities, or weight loss were noticed. We emphasize that the AE used in this study is a chemically defined, water–alcoholic extract of *Withania somnifera*, standardized to either 1.5% or 8.0% total withanolides. These formulations were analyzed and quantified using high-performance liquid chromatography (HPLC) following the United States Pharmacopeia (USP) guidelines, which specify seven characteristic chromatographic peaks for withanolide quantification. This standardization ensures consistency and enhances the interpretability and reproducibility of the results, addressing limitations seen in previous studies using non-standardized commercial extracts.

SD-induced chronic stress significantly reduced body weight and altered serum glucose, AST, and BUN levels, indicating metabolic disturbances in both liver and kidney function. These findings align with the understanding that chronic stress enhances catabolic activity [23], hepatic gluconeogenesis [24], and renal dysfunction [25]. It may explain increased serum glucose, AST levels, and lowered BUN levels due to SD. While all AE-treated groups showed improvement, the high-dose 8% AE formulation was particularly effective in normalizing these biochemical parameters, consistent with previous reports on its hepatoprotective and renoprotective actions [26]. This effect was likely related to AE’s withanolide content, which previously led to a significant decrease in serum bilirubin, ALP, AST, and ALT levels [27].

Stress led to a marked increase in serum corticosterone, CRH, and ACTH levels, reflecting activation of the HPA axis. These results are consistent with prior studies demonstrating HPA axis hyperactivity under SD-related stress conditions [28]. The ability of AE to lower cortisol levels has been previously reported and is thought to be linked to its adaptogenic properties, primarily attributed to bioactive compounds such as withaferin A, as well as its calming and sleep-promoting effects [29]. Moreover, withaferin A can potentiate the anti-stress effect of acylsterylglucosides, namely, sitoindoside VII and sitoindoside VIII. In addition, their combination exhibited significant anti-stress activity [30]. Additionally, glycowithanolides, sitoindoside IX, and sitoindoside X produced significant anti-stress activity in albino mice and rats [31]. In this study, AE—particularly at higher doses—significantly reduced the elevated stress hormone levels, suggesting its effectiveness in modulating excessive HPA axis activity [32].

While SD may increase serotonin release, this response is often independent of stress [33]. In contrast, certain stressors—such as immobility—have been shown to suppress serum serotonin levels in rats [34,35,36]. Sahin et al. [37] demonstrated that caffeine-induced SD impairs both serotonergic and dopaminergic signaling, as evidenced by reductions in serum serotonin and dopamine. Similarly, in the present study, SD-induced stress significantly lowered serotonin and dopamine levels, suggesting disruption of neurotransmitter homeostasis. AE may modulate neurotransmitter activity partly due to its antioxidant and anti-inflammatory effects; however, certain bioactive compounds like withanolides could also directly affect neurotransmitter pathways. Withanolide A has been demonstrated to bind to serotonin receptors and serotonin transporters in both humans and Caenorhabditis elegans through molecular investigations [38]. A methanolic extract of ashwagandha roots (rich in Withaferin and sitoindosides VII-X) was found to inhibit the effects of morphine and ethanol on the dopamine-producing neurons in the ventral tegmental area of the rodent brain [39]. Treatment with AE effectively reversed these declines, underscoring its role in stabilizing neurotransmitter balance. These findings are in agreement with Dawane et al. [34], who reported that AE dose-dependently improved serotonin levels in rats subjected to immobility stress, supporting the current evidence of AE’s neuromodulatory and adaptogenic effects.

The administration of AE exhibited a clear dose-dependent effect in reducing MDA levels, a marker of lipid peroxidation and oxidative stress, in the serum, liver, and brain. The most pronounced reduction was observed at the highest tested dose (11 mg/kg of 8% ashwagandha), suggesting strong antioxidant efficacy. Alongside this, AE, particularly in its high-dose formulation, significantly enhanced the activity of key antioxidant enzymes (SOD, CAT, GSH-Px) and increased TAC in the brain tissue of sleep-deprived rats. The potent antioxidant effects of AE are likely attributed to its rich content of flavonoids, alkaloids, steroidal lactones, saponins, phenolic compounds, withanolides, and other phytochemicals that help mitigate reactive oxygen species (ROS), repair oxidative cellular damage, and reduce lipid peroxidation [40]. These findings are consistent with those of Suganya et al. [41], who reported that AE significantly boosts antioxidant enzyme levels while decreasing oxidative damage and lipid peroxidation in sleep-deprived rats. The bioactive components, sitoindosides VII–X and withaferin A, are considered key contributors to these effects.

Oxidative stress has been closely linked to cognitive deficits, particularly in memory-related tasks [22]. In the current study, rats exposed to SD-induced stress showed impaired performance in the MWM test, as evidenced by fewer entries into the target quadrant and delayed platform acquisition. These impairments are consistent with prior research suggesting that increased oxidative stress in the brain, particularly in the hippocampus, is associated with memory dysfunction and anxiety-like behaviors [42].

Treatment with AE, especially at higher doses, significantly improved spatial memory performance in stressed rats. This cognitive enhancement is likely due to the combined neuroprotective, antioxidant, and anxiolytic properties of AE. The hydrophobic core of β-amyloid 1–42 was shown to interact in the form of oligomers with withanolide A, withanolide B, withanoside IV, withanoside V, and sominone. As a result, further interaction with monomers was prevented, leading to reduced aggregation [43,44]. Specifically, compounds such as withanolide A [45] and withanoside IV may support synaptic function and promote neurogenesis, contributing to improved learning and memory [44]. These results are in agreement with Gladen-Kolarsky et al. [46], who demonstrated that AE improved spatial memory in a dose-dependent manner and correlated these improvements with enhanced brain antioxidant activity in an Alzheimer’s disease mouse model.

The present study highlights the profound impact of chronic stress induced by SD on brain function, mainly through its detrimental effects on neurotrophic factors and inflammatory markers. Key neurotrophic factors essential for neuroplasticity, synaptic connectivity, and neuronal survival, including NCAM, BDNF, NGF, and GAP-43, were significantly downregulated in the brains of SD-exposed rats. Concurrently, levels of ICAM-1, a pro-inflammatory marker associated with neuroinflammation and potential neurodegeneration, were markedly elevated. AE treatment effectively counteracted these alterations, particularly at higher doses. Previous in vitro studies have shown AE’s potential to promote neurodifferentiation in glioma cells [47] and suppress cell migration in neuroblastoma models [48], supporting its neurodegenerative properties. Withanoside IV can prevent Aβ(25–35)-induced axonal, dendritic, and synaptic losses and memory deficits in mice [44]. In line with these findings, the current study observed an upregulation of NCAM in AE-treated animals, suggesting a reversal of SD-induced suppression.

Mechanistic insights from earlier studies further support AE’s neurotrophic potential. Konar et al. [17] demonstrated that AE enhances BDNF expression by modulating cyclic AMP response element-binding protein (CREB) activity and increasing intracellular calcium levels. Similarly, Kim et al. [35] reported that a high dose of AE containing withanolide A elevated BDNF levels in a chronic stress-induced depression model, consistent with our observations. In mice that were treated for spinal cord injury, the expression of BDNF was significantly increased by withaferin A [49]. The neuroprotective effect of BDNF is dependent on GAP-43 activity, which is essential for neuroprotection and neuronal plasticity [50]. Withaferin A- and withanone-treated neuroblastoma cells may exhibit GAP-43 upregulation [17]. Our study, aligned with the findings of [17], demonstrated that AE could enhance the production of brain GAP-43 in memory-impaired mice.

A molecular docking study by Mitra et al. [51] explained the NGF-promoting effect of AE. The study revealed that AE might be acting NGF-mimetic due to interacting with TrkA [51]. Withaferin A-related anti-inflammatory properties of AE [35] can suppress tumor necrosis factor-α-induced expression of nuclear factor-κB and their lower cascade, ICAM-1 [52]. This mechanism may explain the suppressive effect of AE on ICAM-1 in the brain in SD rats.

According to Park et al. [14], AE may improve sleep by increasing the levels of GABA in the brain and boosting the protein levels of GABA_A_, GABA_B1_, and serotonin receptors through the GABAergic system. Biochemical studies suggested that AE’s GABA-mimetic activity originated from some withanolide derivatives (not from withaferin A and withanolide A), which had a GABA-mimetic activity [53]. Sitoindosides VII and VIII, in particular, have been implicated in producing anxiolytic and anti-stress effects, likely by potentiating GABA receptor activity, which leads to reduced neuronal excitability and anxiolysis [54]. This GABA-mimetic action is crucial in mediating ashwagandha’s calming and neuroprotective properties. In a mouse restraint stress model, AE prevented a stress-induced decrease in hippocampal serotonin levels, similar to the present findings [55]. The serotonergic activity of AE was likely associated with the regulation of mRNA expression of serotonin receptors and transporters by withanolide A [38]. Additionally, this GABA-mimetic and serotonergic activity can be improved by administering an elevated dose of AE [14]. In this study, the observed suppression of GABA and serotonin receptors in SD rats may have been mitigated by AE’s dose-dependent GABA-mimetic and serotonergic effects. The combined antioxidant properties of AE and its GABAergic modulation may have synergistically enhanced its neuroprotective and cognitive effects by reducing oxidative stress and improving GABA receptor function. This dual action likely protected neuronal integrity and contributed to improved cognitive performance.

While the present study provides compelling evidence for the neuroprotective, antioxidant, and cognitive-enhancing effects of ashwagandha extract (AE) under chronic stress conditions, several limitations should be acknowledged. First, the absence of a positive control group, such as a standard therapeutic agent (e.g., benzodiazepines, antidepressants, or known adaptogens), represents a limitation. The primary aim of this study was to evaluate the dose-dependent efficacy of AE formulations standardized to specific withanolide concentrations (1.5% and 8.0%), rather than to directly compare AE’s effects with those of established pharmacological treatments. Nonetheless, including a positive control group in future studies would strengthen the comparative value of the findings and help position AE more precisely within the therapeutic landscape of stress-related disorders. Second, the study was conducted solely on male rats, which limits the generalizability of the results to both sexes. Given the known hormonal and neurochemical differences between males and females, it is possible that females may exhibit distinct responses to AE treatment. Additionally, biochemical analyses were conducted on whole-brain homogenates, which precludes the identification of region-specific effects. This is particularly important considering that neurotransmitter receptors and neurotrophic factors exhibit region-dependent expression and function; for example, presynaptic receptors are more prominent in the midbrain, while postsynaptic receptors are abundant in the cortex and hippocampus. Future studies should include region-specific assessments to elucidate the precise neural substrates and molecular pathways modulated by AE. Furthermore, cognitive performance in this study was evaluated using a single behavioral test, which may not fully capture the multifaceted nature of cognition. Employing a comprehensive battery of behavioral assays in future research would enhance the robustness and interpretability of the cognitive outcomes.

## 5. Conclusions

The findings of this study indicate that water-soluble AE, particularly at higher doses, may enhance spatial memory and cognitive performance—likely due to improved bioavailability of its active phytochemicals, especially withanolides. In rats exposed to SD-induced stress, these cognitive benefits were associated with increased brain antioxidant capacity and the GABA-mimetic effects of AE. These results highlight the potential of high-dose AE as a therapeutic option for managing cognitive impairments in clinical contexts. AE may also be beneficial in other models of cognitive impairment, such as aging-related decline and neurodegenerative disorders (e.g., Alzheimer’s or Parkinson’s disease). However, further preclinical investigations and well-designed clinical trials are essential to confirm and extend these observations.

## Figures and Tables

**Table 1:** Effects of different doses of ashwagandha on body weight (BW, g) and serum biochemical parameters (mg/dL) in sleep-deprived (SD) rats (n = 7).

Items	C	WP	SD	SD + A1	SD + A2	SD + A3	SD + A4
Initial BW, g	235.30 ± 5.51	232.90 ± 4.16	233.90 ± 6.116	231.90 ± 5.587	230.70 ± 5.768	233.00 ± 3.823	234.70 ± 5.172
Final BW, g	297.00 ± 4.99 ^a^	293.90 ± 3.06 ^a^	234.6 ± 3.54 ^c^	244.90 ± 6.32 ^bc^	250.1 ± 5.36 ^bc^	253.3 ± 7.22 ^bc^	258.6 ± 5.08 ^b^
Glucose	97.23 ± 2.57 ^d^	101.1 ± 3.66 ^d^	146.40 ± 2.78 ^a^	136.1 ± 3.04 ^ab^	128.2 ± 1.83 ^bc^	130.7 ± 1.56 ^bc^	121.9 ± 2.42 ^c^
Cholesterol	67.22 ± 1.97	66.96 ± 2.29	65.13 ± 1.21	64.59 ± 1.52	66.74 ± 1.36	67.35 ± 2.07	65.26 ± 2.72
Triglyceride	73.93 ± 2.65	74.39 ± 2.40	75.98 ± 1.97	73.96 ± 1.80	73.76 ± 2.06	74.45 ± 2.86	70.01 ± 1.85
AST, U/L	115.10 ± 1.97 ^ab^	116.00 ± 1.90 ^ab^	122.90 ± 2.25 ^a^	115.40 ± 2.64 ^ab^	112.70 ± 1.57 ^b^	114.00 ± 1.78 ^ab^	110.40 ± 2.15 ^b^
ALT, U/L	83.71 ± 2.18	85 ± 2.16	89.71 ± 1.95	86.14 ± 2.40	87 ± 2.29	84.14 ± 1.68	81.86 ± 2.20
BUN	31.07 ± 0.92 ^a^	30.72 ± 0.92 ^a^	23.1 ± 0.79 ^c^	24.29 ± 0.41 ^bc^	24.55 ± 0.53 ^bc^	25.31 ± 0.41 ^bc^	26.98 ± 0.27 ^b^
Creatinine	0.61 ± 0.016	0.60 ± 0.015	0.62 ± 0.021	0.58 ± 0.019	0.58 ± 0.017	0.56 ± 0.022	0.56 ± 0.018

Data are expressed as means ± SEM. Data were analyzed using one-way ANOVA followed by Tukey’s post hoc test for multiple comparisons. Different superscript letters (a–d) in the same row indicate statistically significant differences between groups (*p* < 0.05). Groups not sharing the same letter are significantly different. C: control, WP: wide platform, SD: sleep deprivation, SD + A1: SD + ashwagandha 1.5% (15 mg/kg), SD + A2: SD + ashwagandha 1.5% (30 mg/kg), SD + A3: SD + ashwagandha 8% (5.5 mg/kg), SD + A4: SD + ashwagandha 8% (11 mg/kg). ALT: alanine transaminase, AST: aspartate transaminase, BUN: blood urea nitrogen.

## Data Availability

The original contributions presented in this study are included in the article/Supplementary Material. Further inquiries can be directed to the corresponding author(s).

## Supplementary Materials

The following supporting information can be downloaded at https://www.mdpi.com/article/10.3390/biom15050710/s1, Figure S1: Full immunoblots related to Figure 5 in the main text. Figure S2: Full immunoblots related to Figure 6 in the main text.
