# Supplementary material for: A Novel Ashwagandha (Withania somnifera) Formulation Mitigates Sleep Deprivation-Induced Cognitive Impairment and Oxidative Stress in a Rat Model
Source: Biomolecules. 2025 May 12;15(5):710. doi: 10.3390/biom15050710 (PMC12109121; doi:10.3390/biom15050710)
Supplement: Supplementary file 1 [file biomolecules-15-00710-s001.zip › biomolecules-3596427-supplementary.pdf]

Supplementary File

A Novel Ashwagandha (Withania somnifera) Formulation Mitigates Sleep Deprivation-Induced Cognitive Impairment and Oxidative Stress in a Rat Model

Besir Er[1], Busra Ozmen[2], Emre Sahin[3], Cemal Orhan[2],Nurhan Sahin[2], Abhijeet A. Morde[4], Muralidhara Padigaru[4] and Kazim Sahin [2,*]

[1] Department of Biology, Faculty of Science, Firat University, Elazig 23119, Turkey; ber@firat.edu.tr

[2] Department of Animal Nutrition, Faculty of Veterinary Medicine, Firat University, Elazig 23119, Turkey; busraagzikucuk01@gmail.com (B.O.); corhan@firat.edu.tr (C.O.); nsahin@firat.edu.tr (C.O.).

[3] Department of of Animal Nutrition, Faculty of Veterinary Medicine, Bingol University, Bingol 12100, Turkey; esahin@bingol.edu.tr

[4] Research and Development, OmniActive Health Technologies Co., Ltd., Mumbai 400013, India; a.morde@omniactives.com (A.A.M.); m.padigaru@omniactives.com (M.P.)

* Correspondence: ksahin@firat.edu.tr; Tel.: +90-532-747-3506 or +90-424-237-0000 (ext. 3938)

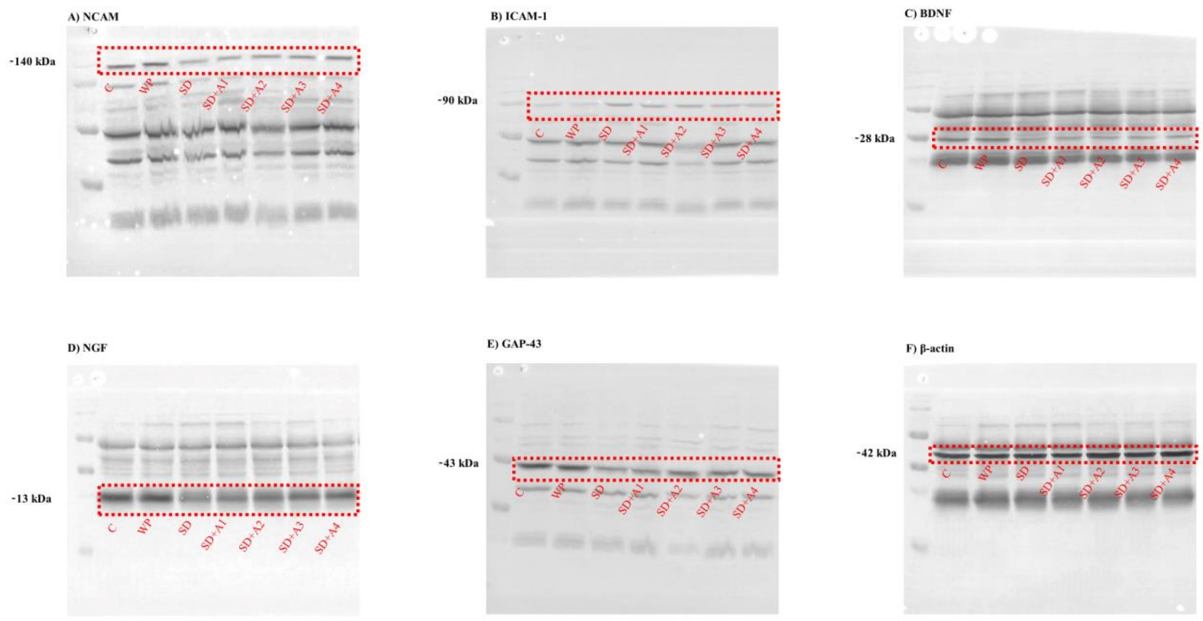

Figure S1. Full immunoblots related to Figure 5 in the main text. The effects of different doses of ashwagandha on brain tissue protein levels of NCAM (A), ICAM-1 (B), BDNF (C), NGF (D), and GAP-43 (E) in sleep-deprived rats are shown. The densitometric analysis of the relative intensity of Western blot bands, normalized to β-actin for ensuring equal protein loading, was performed relative to the control group (F). Red dotted rectangles highlight the results presented in Figure 5 of the main text. Molecular weight (M.W.) markers are shown in kilodaltons (kDa).

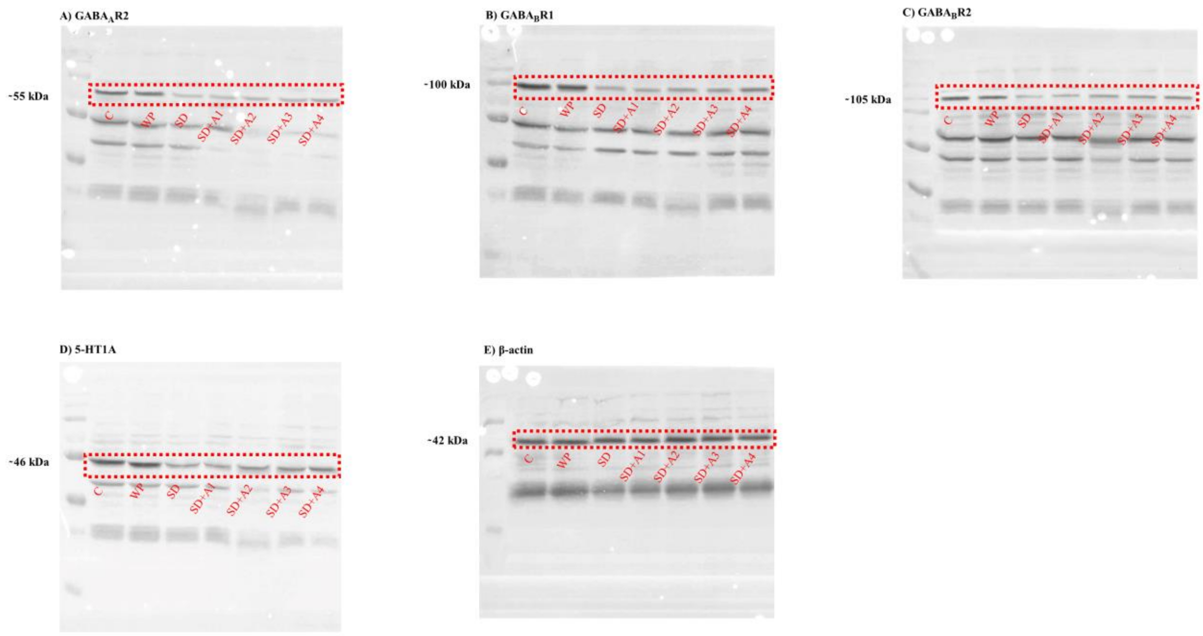

Figure S2. Full immunoblots related to Figure 6 in the main text. The effects of different doses of ashwagandha on brain tissue protein levels of GABA$_A$R2 (A), GABA$_B$R1 (B), GABA$_B$R2 (C), and 5-HT1A (D) in sleep-deprived rats are shown. The densitometric analysis of the relative intensity of Western blot bands, normalized to β-actin for ensuring equal protein loading, was performed relative to the control group (E). Red dotted rectangles highlight the results presented in Figure 5 of the main text. Molecular weight (M.W.) markers are shown in kilodaltons (kDa).
